# Early Age-Related Functional Connectivity Decline in High-Order Cognitive Networks

**DOI:** 10.3389/fnagi.2016.00330

**Published:** 2017-01-10

**Authors:** Tali Siman-Tov, Noam Bosak, Elliot Sprecher, Rotem Paz, Ayelet Eran, Judith Aharon-Peretz, Itamar Kahn

**Affiliations:** ^1^Cognitive Neurology Institute, Rambam Health Care CampusHaifa, Israel; ^2^Department of Neuroscience, Ruth and Bruce Rappaport Faculty of Medicine, Technion – Israel Institute of TechnologyHaifa, Israel; ^3^Laboratory of Clinical Neurophysiology, Ruth and Bruce Rappaport Faculty of Medicine, Technion – Israel Institute of TechnologyHaifa, Israel; ^4^Department of Neurology, Rambam Health Care CampusHaifa, Israel; ^5^Department of Diagnostic Imaging, Rambam Health Care CampusHaifa, Israel

**Keywords:** aging, brain networks, functional connectivity, lifespan, MRI, resting state

## Abstract

As the world ages, it becomes urgent to unravel the mechanisms underlying brain aging and find ways of intervening with them. While for decades cognitive aging has been related to localized brain changes, growing attention is now being paid to alterations in distributed brain networks. Functional connectivity magnetic resonance imaging (fcMRI) has become a particularly useful tool to explore large-scale brain networks; yet, the temporal course of connectivity lifetime changes has not been established. Here, an extensive cross-sectional sample (21–85 years old, *N* = 887) from a public fcMRI database was used to characterize adult lifespan connectivity dynamics within and between seven brain networks: the default mode, salience, dorsal attention, fronto-parietal control, auditory, visual and motor networks. The entire cohort was divided into young (21–40 years, mean ± SD: 25.5 ± 4.8, *n* = 543); middle-aged (41–60 years, 50.6 ± 5.4, *n* = 238); and old (61 years and above, 69.0 ± 6.3, *n* = 106) subgroups. Correlation matrices as well as a mixed model analysis of covariance indicated that within high-order cognitive networks a considerable connectivity decline is already evident by middle adulthood. In contrast, a motor network shows increased connectivity in middle adulthood and a subsequent decline. Additionally, alterations in inter-network interactions are noticeable primarily in the transition between young and middle adulthood. These results provide evidence that aging-related neural changes start early in adult life.

## Introduction

Aging has long been suggested to be accompanied by cognitive decline, even in the absence of dementia or other neurological insults. This decline is more pronounced in cognitive abilities such as processing speed, working memory, and encoding new information into episodic memory, whereas other capacities, particularly semantic knowledge, and emotional processing, seem to remain relatively stable along the adult lifespan (Park et al., 2002; Buckner, 2004; Hedden and Gabrieli, 2004; Park and Payer, 2006; Salthouse, 2009). The precise determination of age-related cognitive changes remains a challenge due to high variability across cognitive skills and research designs. Largely, a monotonous, linear to a first approximation, decline has been reported, starting in the third decade (Buckner, 2004; Park and Reuter-Lorenz, 2009), with some cognitive abilities exhibiting an accelerated deterioration in the seventh decade (Hedden and Gabrieli, 2004).

Age-associated cognitive impairment has frequently been linked to structural modifications in both gray matter (GM) and white matter (WM) (Buckner, 2004; Madden et al., 2009; Bergfield et al., 2010; Giorgio et al., 2010; Thambisetty et al., 2010), though temporal characteristics of these changes and the exact relations with cognitive performance have not been defined (Ferreira et al., 2014). GM volume loss has typically been reported as linear, beginning early in life (Good et al., 2001; Ge et al., 2002; Giorgio et al., 2010; Chen et al., 2013) and only few studies indicated non-linear trends (Terribilli et al., 2011; Lebel et al., 2012; Ziegler et al., 2012). Conversely, WM volume dynamics has frequently been described as non-linear with an inverted-U trajectory over the entire lifespan peaking in the fourth to fifth decade (Giorgio et al., 2010; Chen et al., 2013). Further, fractional anisotropy (FA), a diffusion tensor imaging (DTI) index thought to reflect WM integrity, was shown to deteriorate earlier than WM volume (Salat et al., 2005; Giorgio et al., 2010), quickly after peaking between 20 and 42 years of age (Lebel et al., 2012). Interestingly, a few DTI studies reported a considerable FA decrement in the transition between early and middle adulthood, whereas no significant difference was observed between middle-aged and older adults (Salat et al., 2005; Giorgio et al., 2010).

In addition to structural modifications, alterations in neurotransmitter function have been described in healthy aging, particularly reductions in the binding potential of monoaminergic transmitters (Hedden and Gabrieli, 2004; Park and Reuter-Lorenz, 2009; Bäckman et al., 2010). Age-related cognitive deficits have been most associated with dopaminergic dysfunction (Volkow et al., 1998; Bäckman et al., 2000, 2006; Erixon-Lindroth et al., 2005), though the exact trajectory of dopaminergic decline is still disputed with both linear and non-linear reductions documented (Bäckman et al., 2006). Of note are a few reports highlighting a dramatic decline by midlife in the availability of dopamine receptors (Antonini et al., 1993), dopamine transporters (Mozley et al., 1996), and serotonin receptors (Sheline et al., 2002); yet, these are awaiting confirmation by larger and wider age-range studies.

Importantly, several other neurobiological measures potentially related to cognitive aging are greatly modified by middle-age. For instance, deficits in adult dentate and subventricular zone neurogenesis, considered to play a role in aging, were reported as already established in middle-aged animals (Kuhn et al., 1996; Hamilton et al., 2013). The levels of brain-derived neurotrophic factor (BDNF), phosphorylated cAMP response element binding protein (p-CREB) and neuropeptide Y (NPY), recognized as positive regulators of dentate neurogenesis and memory function, significantly declined as early as middle-age in the rat hippocampus (Hattiangady et al., 2005). Moreover, age-related synaptic plasticity changes, such as long-term potentiation deficits, were evident in middle-aged animals (Rex et al., 2005; Lynch et al., 2006). In humans, signal pathway activation (e.g., ERK-, JNK-, mTOR-, MAPK-, mitochondrial apoptosis, and caspase cascade) in fibroblasts derived from young, middle-aged and old healthy adults as well as Progeria patients showed more pronounced alterations in the transition between the young and middle-aged groups than among other groups (Aliper et al., 2015). Further, a set of genes related particularly to synaptic plasticity, vesicular transport and mitochondrial function showed reduced expression in the frontal cortex after age 40 (Lu et al., 2004). Collectively, this evidence from animal models and humans implies that some age-related decline may present in middle-age.

The timing of age-related structural modifications was reported to vary across brain regions with earlier deterioration in high-order association areas relative to primary sensory and motor cortices (Raz, 2000). Association regions are known to develop relatively late, both ontogenetically and phylogenetically, compared to primary sensory and motor areas (Tau and Peterson, 2010; Fjell et al., 2014); therefore age-related brain changes were suggested to follow the “last-in-first-out” rule (Raz, 2001). Today, brain aging is thought to involve not only regional dysfunction but also alterations in the communication between remote brain areas (Andrews-Hanna et al., 2007; Antonenko and Floel, 2014). In addition to WM structural connections evaluated by means of, for example, DTI, functional connections between distinct GM regions are also explored. Analysis of coherent spontaneous fluctuations in the fMRI blood oxygenation level-dependent (BOLD) signal, termed intrinsic functional connectivity MRI (fcMRI; for reviews, see Fox and Raichle, 2007; Buckner et al., 2013) now allows studying the brain’s connectional architecture across the lifespan. Heretofore, several fcMRI studies have documented age-related reduced connectivity within large-scale intrinsic connectivity networks (ICNs) (Andrews-Hanna et al., 2007; Damoiseaux et al., 2008; Koch et al., 2010; Tomasi and Volkow, 2012; Ferreira and Busatto, 2013), and altered connectivity between ICNs has also been proposed (Onoda et al., 2012; Antonenko and Floel, 2014). However, this literature refers to only a few ICNs, lifespan trajectories of intra-network connectivity decline and differences between networks are not well characterized and results regarding inter-network connectivity changes are variable, with both increases (Meunier et al., 2009; Betzel et al., 2014; Chan et al., 2014; Geerligs et al., 2015; Grady et al., 2016; Spreng et al., 2016) and decreases (Meunier et al., 2009; Allen et al., 2011; Onoda et al., 2012) reported.

The present study aims to fill this gap and extend our view of intra- and inter-network functional reorganization during aging. The authors believe that a comprehensive outlook of the lifetime dynamics of brain functional connectomics is crucial for deciphering the mechanisms underlying brain aging, determining intervention targets and their optimal timing. Herein, an extensive fcMRI cohort of 887 healthy individuals between the ages of 21 and 85 was used to characterize functional connectivity changes across the adult lifespan in seven robust well-established ICNs: the default mode (DMN), salience (SN), dorsal attention (DAN), fronto-parietal control (FPCN), auditory (AN), visual (VN), and motor (MN) networks (Damoiseaux et al., 2006; Van den Heuvel and Hulshoff Pol, 2010; Van Dijk et al., 2010; Yeo et al., 2011; Brier et al., 2012). Consistent with previous lifespan studies (Giorgio et al., 2010; Van Helmond et al., 2010; Park et al., 2013; Ferreira et al., 2015; Kennedy et al., 2015), the cohort was divided into young, middle-aged, and old groups. Consistent with earlier imaging literature (Li et al., 2011, 2013; Montembeault et al., 2012; Belcher et al., 2013; Geerligs et al., 2015) the seven networks were divided into high-order cognitive networks (DMN, SN, DAN, and FPCN), known to involve associative brain areas and play a role in high-order cognition (Van den Heuvel et al., 2009; Menon, 2011; Braga and Leech, 2015) and primary sensory and motor networks (AN, VN, and MN). Differential response of these network groups to aging was assumed based on recent fcMRI reports (Chan et al., 2014; Geerligs et al., 2015) and the “last-in-first-out” theory (Raz, 2001). Taken together, we expected functional connectivity decline by middle-age within high-order cognitive networks, as well as early between-network connectivity alterations, where high-order cognitive networks are involved.

## Materials and Methods

### Participants

Intrinsic functional connectivity MRI data recorded at rest of 887 healthy individuals (age range 21–85 years, 514 females) were obtained from the online dataset of the International Neuroimaging Data-sharing Initiative (INDI), ‘1000 Functional Connectomes’ Project^1^ (Mennes et al., 2013). The entire cohort was subdivided into three groups: young (21–40 years, mean ± SD: 25.5 ± 4.8, *n* = 543, 284 females); middle-aged (41–60 years, 50.6 ± 5.4, *n* = 238, 160 females); and old (61 years and above, 69.0 ± 6.3, *n* = 106, 70 females). Seventeen research sites were included (**Table 1**; Supplementary Figure S1). Each center’s ethics committee approved submission of de-identified data. The institutional review board of Rambam healthcare campus approved the receipt and analysis of these data. Centers including less than 10 participants and data of participants with partial brain coverage were discarded from analysis. Sixteen additional individuals were not included due to excessive head movement. Three hundred and eighteen out of the 887 participants (**Table 2**) were selected for further statistical analysis as detailed below.

**Table 1:** Centers of the 1000 functional connectomes project included in the study; epidemiological and fMRI acquisition information.

Center/Publication year	*n*	M/F	Age range	Scanner	TR (s)	Slices	Time-points^1^	Voxel size (mm^3^)	Eyes	Handedness
Atlanta 2009	28	13/15	22–57	3T	2	20	205	3.4375 × 3.4375 × 4	Open, fixation	Four left-handed
Beijing 2009	119	48/71	21–26	3T	2	33	225	3.12 × 3.12 × 3.6	Closed	Right-handed only
Berlin 2009	26	13/13	23–44	3T	2.3	34	195	3 × 3 × 4	Open, blank screen	Right-handed only
Cambridge 2009	101	40/61	21–30	3T	3	47	119	2 × 2 × 4	Open	Twelve left-handed
Cleveland 2009	26	9/17	24–60	3T	2.8	31	127	4 × 4 × 5.5	Closed	Right-handed only
COBRE 2012	66	47/19	21–65	3T	2	32	150	3 × 3 × 4	NA	One left-handed/1 ambidextrous
Dallas 2009	21	10/11	21–71	3T	2	36	115	3.44 × 3.44 × 4	NA	NA
Leiden 2009	10	10/0	21–27	3T	2.18	38	215	3.44 × 3.44 × 3.44	Closed	Right-handed only
Leiden 2009	11	5/6	21–28	3T	2.2	38	215	3 × 3 × 4	Closed	Right-handed only
Milwaukee 2009	43	14/29	44–65	3T	2	64	175	3.75 × 3.75 × 4	NA	NA
Munich 2009	14	9/5	63–73	1.5T	3	33	72	3.44 × 3.44 × 5	Closed	Right-handed only
New York 2009	32	18/14	22–49	3T	2	39	192	3 × 3 × 3	Open	Right-handed only
NKI-RS 2014	307	97/210	21–85	3T	2.5	38	120	3 × 3 × 3	Open, fixation	Twenty-three left-handed/17 unknown
Orangeburg 2009	17	13/4	25–55	1.5T	2	22	165	3.5 × 3.5 × 5	Closed	Three left-handed
Palo Alto 2009	17	2/15	22–46	3T	2	22	245	3.4375 × 3.4375 × 4.9	NA	Right-handed only
Queensland 2009	18	11/7	21–34	3T	2.1	36	190	3.59 × 3.59 × 3.6	Open	Right-handed only
St. Louis 2009	31	14/17	21–29	3T	2.5	32	127	4 × 4 × 4	Open, fixation	Right-handed only

*^1^Data were acquired with dummy scans to avoid including frames with T1-equilibration effects. COBRE, Center for Biomedical Research Excellence; ICBM, International Consortium for Brain Mapping; NKI-RS, Nathan Kline Institute-Rockland Sample.*

**Table 2:** Epidemiological data of participants selected for statistical analysis.

Center/Publication year	*n*	M	F	Age range	Handedness
Atlanta 2009	7	5	2	23–57	Two left-handed
Beijing 2009	18	7	11	21–26	Right-handed only
Berlin 2009	5	3	2	26–44	Right-handed only
Cambridge 2009	22	8	14	21–30	Three left-handed
Cleveland 2009	3	0	3	53–60	Right-handed only
COBRE 2012	21	15	6	23–65	Right-handed only
Dallas 2009	11	5	6	21–71	NA
Leiden 2009	4	4	0	21–24	Right-handed only
Leiden 2009	2	0	2	21–22	Right-handed only
Milwaukee 2009	24	8	16	47–65	NA
Munich 2009	14	9	5	63–73	Right-handed only
New York 2009	8	4	4	23–45	Right-handed only
NKI-RS 2014	159	46	113	21–85	Fourteen left-handed/5 unknown
Orangeburg 2009	5	5	0	26–55	Right-handed only
Palo Alto 2009	6	0	6	26–46	Right-handed only
Queensland 2009	2	0	2	26	Right-handed only
St. Louis 2009	7	3	4	21–28	Right-handed only
**Total**	**318**	**122**	**196**	**21**–**85**	**19 left-handed/5 unknown**

### Functional Imaging Data Acquisition and Preprocessing

Acquisition data are shown in **Table 1**. Functional images were preprocessed using FSL software (FMRIB Software Library v. 5.0.1, Oxford, UK) and SPM software (Statistical Parametric Mapping software package, Wellcome Department of Imaging Neuroscience, London, UK) following conventional methods as previously described (Kahn et al., 2008; Kahn and Shohamy, 2013). Preprocessing included rigid body correction for motion within and across runs (FSL), normalization to the standard EPI template of the Montreal Neurological Institute (MNI) and compensation for slice-dependent time shifts (SPM). The preprocessed functional data (in atlas space) were then temporally filtered to remove constant offsets and linear trends over each run while retaining frequencies below 0.08 Hz. Data were spatially smoothed using a 4 mm full-width half-maximum Gaussian blur. Sources of spurious or regionally non-specific variance were removed by regression of nuisance variables including six parameters obtained by rigid body head motion correction, the signal averaged over the whole brain (global signal), the signal averaged over the lateral ventricles and the signal averaged over a region centered in the deep cerebral WM. Temporally shifted versions of these waveforms were removed by inclusion of the first temporal derivatives (computed by backward differences) in the linear model. The data was also analyzed without global signal regression (GSR) to evaluate its potential influence on our findings.

### Functional Connectivity Analysis

Seed-based analysis was performed as previously described (Fox et al., 2005; Kahn et al., 2008; Van Dijk et al., 2010) to study four high-order cognitive networks: DMN, SN, DAN, and FPCN as well as three primary sensory and motor networks: AN, VN, and MN. To delineate these networks, the following seed regions were used: left posterior cingulate cortex (LPCC); right frontoinsula (RFI); right intraparietal sulcus (RIPS); right superior parietal cortex (RSP); left auditory cortex (LAC); right visual cortex (RVC); and left motor cortex (LMC). Each seed was defined as a 6 mm radius sphere centered on previously published foci (**Table 3**). Correlation maps were produced by extracting the time course from each of the above seeds and computing the Pearson correlation coefficient (*r*) between this time course and the time course of each voxel across the whole brain. SPM8 software was used to compute statistical maps of each network across participants. Maps of a young age group (458 participants, 21–30 years) were used to identify peak coordinates of additional regions of interest (ROIs) representing each network. All regions were defined as 6 mm radius spheres around the peak coordinate (**Table 3**). For statistical tests, the Fischer’s *r*-to-*z* transformation (*z(r)* = 0.5 ln[(1 + *r*)/(1 -*r*)]) was applied. Using MATLAB version R2012b (Mathworks, Natick, MA, USA), node pair correlation matrices were computed for the three age groups: young (21–40 years), middle-aged (41–60 years), and old (61 years and above). Additionally, between age group difference matrices were calculated for the young vs. the middle-aged group and the middle-aged vs. the old group. These matrices exhibit the age group difference in connectivity strength [*z(r)* value] for each node pair. Correction for multiple comparisons was carried out using the false discovery rate (FDR) procedure (Benjamini and Yekutieli, 2001) implemented in MATLAB with no assumptions about test dependency and at an FDR level of 0.05. Group difference maps for the DMN and MN were computed using the two-sample *t*-test implemented in SPM8. Three significant ROIs were chosen for each of the four contrasts to present *z(r)* values of ROI-to-network seed (LPCC or LMC) across participants within each age group. Regions were defined as 4 mm radius spheres around the peak coordinate. Box-and-whisker plots were drawn using MATLAB.

**Table 3:** Network regions of interest.

Network	ROI name	Abbreviation	MNI coordinates		
			*x*	*y*	*z*
DMN	L posterior cingulate cortex*^1^*	LPCC	-8	-56	26
	R posterior cingulate cortex	RPCC	6	-52	24
	L angular gyrus	LAngular	-44	-68	36
	R angular gyrus	RAngular	50	-62	32
	L anterior medial prefrontal cortex	LamPFC	-2	54	-8
	L lateral temporal cortex	LLTC	-60	-10	-20
	R lateral temporal cortex	RLTC	62	-8	-22
SN	R Frontoinsula*^2^*	RFI	35	24	5
	L Frontoinsula	LFI	-32	20	6
	R anterior cingulate cortex	RACC	6	16	42
	R dorsolateral prefrontal cortex	RDLPFC	38	48	26
	L dorsolateral prefrontal cortex	LDLPFC	-38	42	22
	R supramarginal gyrus	RSMG	62	-34	40
DAN	R intraparietal sulcus*^3^*	RIPS	22	-58	54
	L intraparietal sulcus	LIPS	-22	-58	56
	R posterior intraparietal sulcus	RpostIPS	28	-74	34
	L posterior intraparietal sulcus	LpostIPS	-22	-76	34
	R frontal eye field	RFEF	26	-4	52
	R middle temporal gyrus	RMTG	50	-60	-8
	L middle temporal gyrus	LMTG	-48	-66	-4
FPCN	R superior parietal cortex*^4^*	RSP	53	-49	47
	L superior parietal cortex	LSP	-54	-50	48
	R middle frontal gyrus	RMFG	44	30	40
	L middle frontal gyrus	LMFG	-44	28	38
	R dorsomedial prefrontal cortex	RdmPFC	4	30	44
	R dorsal posterior cingulate cortex	RdPCC	4	-36	44
	R frontal pole	RFP	36	58	0
AN	L primary auditory cortex*^4^*	LAC	-64	-28	13
	R primary auditory cortex	RAC	60	-24	14
	L anterior cingulate cortex	LACC	-4	2	44
	R anterior cingulate cortex	RACC	2	-4	48
	L anterior lateral sulcus	LantLatSul	-56	0	-2
	R anterior lateral sulcus	RantLatSul	60	0	2
VN	R primary visual cortex*^5^*	RVC	7	-76	10
	L primary visual cortex	LVC	-7	-76	10
	R lateral geniculate nucleus	RLGN	22	-26	-6
	L lateral geniculate nucleus	LLGN	-22	-28	-6
MN	L primary motor cortex*^3^*	LMC	-36	-25	57
	R primary motor cortex	RMC	40	-26	56
	Supplementary motor area	SMA	-2	-20	54
	L insula	LInsula	-36	-18	16
	R insula	RInsula	38	-16	16

*MNI coordinates of regions of interest (ROIs) representing each network. The first ROI of each network was drawn from the literature and used as a seed region to delineate the respective network: (1) Andrews-Hanna et al., 2010. (2) Seeley et al., 2009. (3) Van Dijk et al., 2010. (4) Brier et al., 2012. (5) Dai et al., 2012. L, left; R, right.*

### Head Motion Correction

As head motion is widely considered a significant confound in fcMRI (Power et al., 2012; Van Dijk et al., 2012) and in view of previous reports suggesting increased head movement with aging (D’Esposito et al., 1999; Van Dijk et al., 2012), additional head motion parameters were calculated for each participant as proposed by Power et al. (2012) : (1) Framewise displacement (FD), which represents head displacement from volume to volume, was computed as the sum of the first derivative of the six rigid-body motion parameters estimated during standard volume realignment; (2) Delta variation signal (DVARS), which represents the change in BOLD signal intensity from one frame to the next, was computed as the root mean square average of the first derivative of fMRI signals across the entire brain. A standardized version of DVARS was applied according to Nichols (2013). Averaged FD values were below 0.5 mm for all participants. Averaged standardized DVARS values were between 0.7 and 1.5. Each individual’s averaged FD and DVARS values were included in the statistical analysis as described below.

### Cortical Thickness Analysis

To correct for a potential effect of age-related cortical atrophy, we estimated cortical thickness using the Freesurfer image analysis suite, version 5.2.0^2^. Freesurfer uses intensity and continuity information from the entire three dimensional magnetic resonance volume in segmentation and deformation procedures to produce representations of cortical thickness (Dale et al., 1999). Three-dimensional T1-weighted magnetization-prepared rapid acquisition with gradient echo (MPRAGE) scans were used for analysis. Cortical reconstruction and volumetric segmentation were performed for each participant and the boundaries between GM and WM were delineated. Cortical thickness was defined as the closest distance from the GM/WM border to the GM/cerebrospinal fluid border at each vertex along the tessellated surface (Fischl and Dale, 2000). Both the ROIs and each participant’s surface representation of the cortex were registered to *fsaverage*, the freesurfer template subject. The measure of cortical thickness was averaged across the vertices that comprise each ROI representation over each participant’s surface. These values were then averaged across all ROIs within a network. Each participant’s mean cortical thickness per network was included in the statistical analysis as described below.

### Statistical Analysis

Regression modeling was utilized to examine influences on functional connectivity. Averaged *z(r)* values of node pair correlations within each network were calculated for each participant. These connectivity scores were then analyzed as the dependent variable by a mixed model analysis of covariance (ANCOVA). This modeling method allowed us to examine the effect of a number of factors, and their interactions, while accounting for (1) the effects of covariates on connectivity; (2) systematic differences between test centers; and (3) the influence of individual subjects who each provided data on all networks. Specifically, network, age group, gender and all their factorial interactions served as fixed independent factors; FD, DVARS and cortical thickness served as covariates; test center, assumed to be a random sample from the population of all possible test centers, served as a random effect; and participant, presumed to be part of a random sample of all possible subjects, served as a random factor, necessarily nested in age group, gender and center (utilization of both fixed and random factors makes the model “mixed”). The factor ‘age group’ had three levels: young (21–40 years), middle-aged (41–60 years), and old (61 years and above), to fit our hypotheses. Gender was included as a factor following prior literature suggesting a gender effect on functional connectivity in general (Allen et al., 2011; Tomasi and Volkow, 2012) and on age-related connectivity changes in particular (Scheinost et al., 2015). We assumed that variability in scanning parameters across centers (scanner magnetic field strength, imaging protocol, image geometry, and scan time) would be sufficiently accounted for by treating center as a random effect. Since an unbalanced model with many factorial interactions could be problematic, 106 participants were randomly selected from the young and middle-aged groups to match the number of individuals within the older age group (**Table 2**; Supplementary Figure S1). SAS version 9.3, JMP version 11 (both SAS Institute, Cary, NC, USA) and Microsoft Excel 2013 (Microsoft Corp., Redmond, WA, USA) were used for data analysis.

Inspection of ANCOVA diagnostic plots, particularly residual information, did not indicate violation of required normality assumptions, so no transformations were applied to the data. Although not a focus of our research, a full treatment of models under mixed modeling generally examines the patterns of relationships (covariance structures) between elements of random effects; when there is no *a priori* hypothesis for any particular covariance structure, tests of several candidates are run, with the best among them determined by statistical methods, such as information criteria tests. Tests of several candidate covariance structures by SAS PROC MIXED indicated that the variance components covariance structure was most appropriate based on the AICC (corrected Akaike information criterion). This implies that variances associated with each center and each subject were different, and that there was no covariance between either centers or subjects.

The above described model does not provide tests of specific hypotheses of interest regarding the age-group-related connectivity changes. Yet, it does provide the basic information for constructing specific contrasts which are of direct relevance to our hypotheses. Therefore, specific, pre-planned, multiplicity-corrected contrasts were constructed to examine: (1) whether a significant difference emerged within each of the seven networks in the transition from young to middle-aged vs. middle-aged to old; (2) whether these changes in each high-order cognitive network (from young to middle-aged vs. middle-aged to old) were significantly different from equivalent changes in each primary sensory and motor network. The contrasts were multiplicity-corrected (for multiple tests) using the simulation method offered by SAS PROC MIXED. Statistical significance after multiplicity correction was at the level of *p* ≤ 0.05.

## Results

### Temporal Signature of Age-Related Connectivity Decline

**FIGURE 1:**
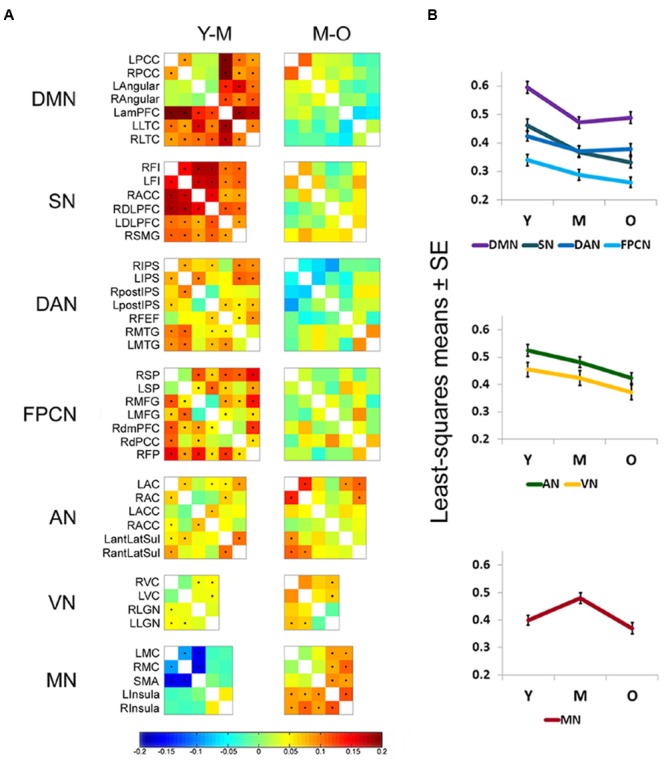
**Early age-related connectivity decline in high-order cognitive networks. (A)** Difference matrices of young vs. middle-aged (Y-M) and middle-aged vs. old (M-O) participants (entire cohort, *n* = 887) are shown for each network. Rows and columns of matrices denote the ROIs that were chosen to represent each network (see **Table 3**). Matrix entries represent the age group difference in connectivity strength [*z(r)* value] for each node pair. Entries marked by a dot survived FDR correction for multiple comparisons. In general, a significant connectivity decline is observed in high-order cognitive networks in the transition between young and middle adulthood. Within the AN and VN significant reductions are observed in some pairs for both transitions, Y to M and M to O. The MN mostly shows early connectivity increments and late connectivity decrements. See **Table 3** for regions full name. **(B)** ANCOVA results of the network × age group interaction [performed on a subset of the entire cohort (*n* = 318)] are presented as least-squares means ± standard error (SE). Connectivity reduction within high-order cognitive networks is more pronounced in the comparison between Y to M than between M to O. The AN and VN show connectivity reduction in both transitions, with a more prominent decline in the transition between M to O. The MN shows an early significant connectivity increment followed by a late significant decrement.

To study connectivity dynamics across the adult lifespan, connectivity matrices were computed for each age group (young, middle-aged, and old) for the following networks: DMN, SN, DAN, FPCN, AN, VN, and MN. Difference matrices were then calculated for each network between the young and middle-aged (Y-M) and the middle-aged and old (M-O) groups. These comparisons yielded similar results for all high-order cognitive networks (DMN, SN, DAN, and FPCN): Y-M matrices mostly showed significant connectivity reductions, while M-O matrices revealed non-significant decreases, or a tendency for increased connectivity in some pairs, particularly within the DMN and DAN (**Figure 1A**). In contrast, the MN typically showed increased connectivity in the transition from young to middle-aged and decreased connectivity in the transition from middle-aged to old. Increased connectivity in the transition to middle-age was particularly noted in the connections between the supplementary motor area and the primary motor cortex bilaterally. Within the AN and VN both comparisons yielded significant decrements.

To account for potential sources of bias such as brain atrophy, head motion and varying acquisition parameters, a mixed-model ANCOVA was conducted on a subset of 318 participants of the entire cohort, after equalizing the number of participants in each age group (**Table 4**). Network and age group showed a highly significant main effect (*p* < 0.0001) and a gender main effect was significant at *p* = 0.05. Network × age group and network × gender interactions both reached significance (*p* < 0.0001 and *p* = 0.01, respectively), but network × age group × gender interaction did not (*p* = 0.38). Least-squares means (LSM) and standard errors (SE) of the network × age group interaction are plotted in **Figure 1B**. These analyses confirmed the findings of the correlation matrices, showing an early decline in connectivity within high-order cognitive networks, predominantly in the DMN and SN. Pre-planned, multiplicity-corrected contrasts of the transition from young to middle-aged against middle-aged to old within each network revealed significant differences for the DMN and MN (**Table 5**). For the DMN, a significant decrement from young to middle-aged was followed by a minor increment from middle-aged to old. The MN showed a completely different pattern of lifelong connectivity changes: an increase from young to middle-age, followed by an equivalent decrease at old age. Contrasts of the transition between the young to middle-aged and middle-aged to old were compared between high-order cognitive networks and primary sensory and motor networks. Pre-planned, multiplicity-corrected comparisons showed a significant difference between the MN and all other high-order cognitive networks (**Table 6**).

**Table 4:** ANCOVA summary.

Effect	Num DF	Den DF	*F-v*alue	*P*_r_ > *F*
Gender	1	310	3.91	0.0488
Network	6	1901	53.19	<0.0001
Network × Gender	6	1874	2.68	0.0135
Age Group	2	325	14.59	<0.0001
Age Group × Gender	2	310	0.36	0.6949
Network × Age Group	12	1874	4.36	<0.0001
Network × Age Group × Gender	12	1871	1.08	0.376
DVARS	1	314	24.85	<0.0001
FD	1	310	6.09	0.0141
Cortical Thickness	1	1873	4.18	0.041

*Summary table of ANCOVA for connectivity. Num DF, numerator degrees of freedom; Den DF, denominator degrees of freedom; *F*-value, the F statistic; *P*_*r*_ > F, the *P-*value associated with the test of this factor.*

**Table 5:** Pre-planned contrasts between age groups within each network.

	Multiplicity-adjusted *P*-values
DMN	**0.034**
SN	0.8786
DAN	0.8681
FPCN	0.9993
AN	0.9999
VN	0.9993
MN	**0.0006**

*A summary of the pre-planned, multiplicity-corrected contrasts of the transition from young to middle-aged against middle-aged to old within each network. Statistically significant values are denoted in bold (*p* < 0.05).*

**Table 6:** Pre-planned contrasts between high-order cognitive networks and primary sensory and motor networks.

	Multiplicity-adjusted P-values
DMN vs. MN	**<0.0001**
DMN vs. AN	0.1039
DMN vs. VN	0.0747
SN vs. MN	**0.0005**
SN vs. AN	0.8835
SN vs. VN	0.8217
DAN vs. MN	**0.0005**
DAN vs. AN	0.8775
DAN vs. VN	0.8118
FPCN vs. MN	**0.0039**
FPCN vs. AN	0.9965
FPCN vs. VN	0.9886

*A summary of the pre-planned, multiplicity corrected contrasts of the transition between the young to middle-aged and middle-aged to old as compared between high-order cognitive networks and primary sensory and motor networks. Statistically significant values are denoted in bold (*p* < 0.05).*

### Spatial Signature of Early Age-Related Connectivity Changes in the DMN and MN

**FIGURE 2:**
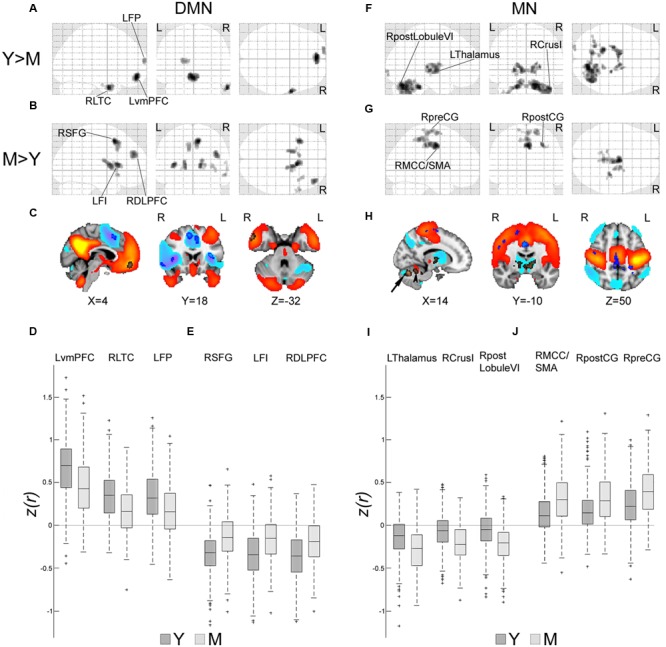
**Spatial signature of early age-related connectivity changes in the DMN and MN.** Age group comparisons of spatial *z(r)*-maps of the DMN and MN were computed by the SPM two-sample *t*-test [*p* < 0.001, family-wise-error (FWE) corrected]. **(A,F)** Glass brains showing regions of decreased connectivity in middle-aged (M) vs. young (Y) participants within the DMN and MN, respectively. **(B,G)** Glass brains showing regions of increased connectivity in M vs. Y within the DMN and MN, respectively. **(C)** Brain maps of the DMN (one sample *t*-test, Y age-group, *p* < 0.001, FWE corrected, warm colors), and DMN anticorrelated network (cool colors). Regions of **(A)** (copper-colored) appear within DMN and reflect reduced connectivity in M vs. Y. Regions of **(B)** (blue-colored) appear within the DMN anticorrelated network, and therefore represent decreased anticorrelation with the LPCC in M vs. Y. **(D)** Box plots of *z(r)* values between LPCC and three representative regions of **(A)** [LvmPFC (-2,52,-12), RLTC (62,2,-30) and LFP (-16,66,16)] across participants of Y and M. Most values are positive and reductions are observed from Y to M. **(E)** Box plots of *z(r)* values between LPCC and three representative regions of **(B)** [RSFG (10,14,52), LFI (-34,20,10) and RDLPFC (42,48,30)]. Most values are negative and increases (reduced anticorrelations) are observed from Y to M. **(H)** Brain maps of the MN (one sample *t*-test, Y age-group, *p* < 0.001, FWE corrected, warm colors) and MN anticorrelated network (cool colors). Regions of **(F)** (copper-colored) mostly appear within the MN anticorrelated network, representing increased anticorrelation with the LMC in M vs. Y. These regions included the thalamus, basal ganglia and posterior cerebellum (arrow). A region within the anterior cerebellum (arrowhead) shows reduced connectivity with the LMC. Regions of **(G)** (blue-colored) appear mainly within or adjacent to the MN and reflect enhanced connectivity in M vs. Y. **(I)** Box plots of *z(r)* values between LMC and three representative regions of **(F)** [LThalamus (-8,-8,0), RCrusI (42,-64,-32) and RpostLobuleVI (16,-70,-26)]. Most values are negative and reductions (increased anticorrelations) are observed from Y to M. **(J)** Box plots of *z(r)* values between LMC and three representative regions of **(G)** [RMCC/SMA (4,-8,44), RpostCG (30,-28,44) and RpreCG (20,-16,70)]. Most values are positive and increases are observed from Y to M. LFI, left frontoinsula; LFP, left frontal pole; LThalamus, left thalamus; LvmPFC, left ventromedian prefrontal cortex; RCrusI, Right crus I of cerebellar hemisphere; RDLPFC, right dorsolateral prefrontal cortex; RLTC, right lateral temporal cortex; RMCC/SMA, right middle cingulate cortex/supplementary motor area; RpostCG, right postcentral gyrus; RpostLobuleVI, right posterior Lobule VI of cerebellar hemisphere; RpreCG, right precentral gyrus; RSFG, right superior frontal gyrus.

To anatomically characterize the unique and opposed effects of age on connectivity within the DMN and MN, a two-sample *t*-test analysis was used to compare network spatial maps of young vs. middle-aged individuals. Within the DMN, reduced connectivity was shown in middle-aged vs. young participants in several regions of the network, primarily the ventromedial prefrontal cortex (vmPFC), the right lateral temporal cortex (LTC), and the left frontal pole (**Figures 2A,C** copper-colored regions and **Figure 2D**). Increased correlation with the LPCC was also observed, mainly within frontal regions bilaterally (**Figures 2B,C** blue-colored regions and **Figure 2E**). These regions were found to be associated with the DMN anticorrelated network (**Figure 2C**, cool colors); therefore, connectivity increments in this case represent reductions in the magnitude of anticorrelation with the LPCC. Within the MN, the comparison between young and middle-aged participants showed decreased connectivity in the latter group in an anterior region of the cerebellum, and increased anticorrelations with regions in the thalamus, basal ganglia and posterior cerebellum (**Figures 2F,H** copper-colored regions and **Figure 2I**). Increased connectivity in middle-aged relative to young individuals was noticed mainly in cortical regions of the MN or border regions adjacent to the MN (**Figures 2G,H** blue-colored regions and **Figure 2J**).

### Early Age-Related Inter-Network Connectivity Changes

**FIGURE 3:**
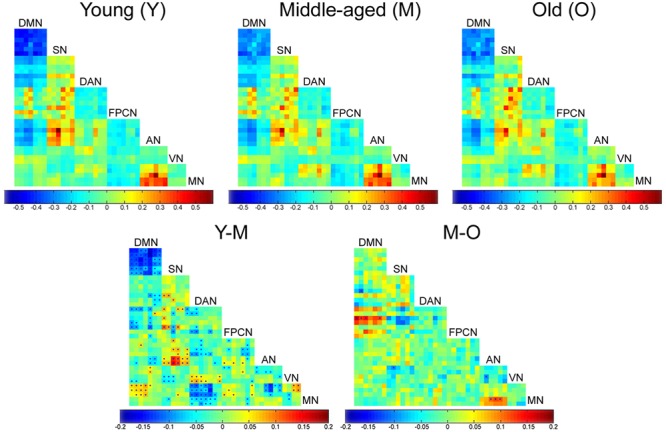
**Age-related inter-network connectivity changes.** Inter-network connectivity matrices of the young (Y), middle-aged (M) and old (O) groups (*upper row*). Rows and columns denote the ROIs that were chosen to represent each network (see **Table 3**). Matrix entries represent connectivity strength between each two nodes (*z(r)* value). Difference matrices of the Y vs. M and M vs. O groups (*lower row*). Matrix entries represent age-group difference in connectivity strength for each node pair. Entries marked by a dot survived FDR correction for multiple comparisons. Most significant age-related changes in inter-network connectivity occurred in the transition between Y and M groups.

Connectivity matrices calculated for the three age groups showed connectivity dynamics not only within networks as described above, but also between networks. Most of the inter-network connectivity changes occurred in the transition between the young and middle-aged groups and involved high-order cognitive networks. Early significant reduced connectivity was found between the DMN and MN, SN and AN, and DAN and VN. Early significant increased connectivity was found between the DAN and MN, DAN and AN, SN and VN, AN and MN, parts of the DMN and FPCN, and parts of the AN and VN. Another early change was a decline in anticorrelations: DMN-SN, DMN-DAN, and DAN-FPCN. Late changes were much less pronounced. Significant alterations were noticed in only 10 out of 752 node pairs, compared with 203 pairs in the transition from young to middle-age. Late changes included reduced connectivity between the DMN and FPCN and AN and MN, along with increased connectivity between the SN and FPCN, SN and VN, and AN and VN (**Figure 3**).

### Global Signal Regression Effect on Study Results

Reanalysis of the data without GSR yielded similar results (Supplementary Figures S2 and S3). As expected, a general increase in correlations within and between networks was evident (Murphy et al., 2009; Van Dijk et al., 2010; Murphy and Fox, 2016), but this did not affect the major observations of the study. Specifically, the within network correlation pattern did not change. Age group difference matrices revealed greater reduction in the transition from Y to M than from M to O for the DMN, SN, and FPCN. The MN showed increased connectivity from Y to M followed by a reduction for O. The AN and VN showed reductions in both transitions, but Y to M reduction in the AN was much less significant relative to results with GSR. The observation of a less significant age-related reduction when avoiding GSR, particularly within the DAN and AN from Y to M, might be related to high correlation between the signal of these networks and the global signal. The global signal has been shown to highly correlate with signals of the visual, auditory and somatosensory networks (Fox et al., 2009). In our study the DAN was positively correlated with these three non-cognitive networks.

Between network correlations appeared to be affected more markedly by GSR removal. However, this mainly stemmed from the expected disappearance of anticorrelations (Fox et al., 2009; Murphy et al., 2009), while the direction of change between age groups was mostly kept, with the exception of the early decrement in DMN-MN and the late increment in AN-VN. Moreover, the finding that most of the inter-network connectivity alterations occur in the transition from young to middle-age rather than from middle-age to old was even accentuated by omitting GSR.

## Discussion

The present findings indicate that functional connectivity (FC) decline in high-order cognitive networks is already evident by middle-age, as expressed by fcMRI measures. The time course of FC alterations within a motor network is characterized by an early increment followed by a decrement. Finally, most inter-network connectivity changes occur in the transition from young to middle adulthood, highlighting that several potentially meaningful FC changes onset early in the adult lifespan.

Growing evidence now suggests that age-related cognitive decline is associated with FC alterations within large-scale brain networks (Andrews-Hanna et al., 2007; Damoiseaux et al., 2008; Ferreira and Busatto, 2013). The DMN has been the focus of much of this literature, most studies reported FC decline with age and associated FC decline with reduced cognitive performance (e.g., Andrews-Hanna et al., 2007; Damoiseaux et al., 2008; Ferreira and Busatto, 2013; Vidal-Piñeiro et al., 2014). More recently, age-related connectivity decreases have also been noted in other ICNs (Andrews-Hanna et al., 2007; Onoda et al., 2012; Tomasi and Volkow, 2012; Ferreira and Busatto, 2013; Geerligs et al., 2015). Importantly, most of the above mentioned studies directly compared young and old adults, while only a few studied young and middle-aged individuals and actually reported diminished FC in middle-adulthood (Bluhm et al., 2008; Allen et al., 2011; Evers et al., 2012). Here, FC decline by middle-age is observed in an extensive cohort of individuals within the DMN, SN, DAN, and PFCN. This early FC decline accords with previously suggested trajectories of cognitive and structural deterioration with aging (Buckner, 2004; Park and Reuter-Lorenz, 2009; Giorgio et al., 2010; Chen et al., 2013). Furthermore, several recent studies explicitly underscored a detectable cognitive decline by middle-age in both humans (Singh-Manoux et al., 2012; Ferreira et al., 2015) and animals (Moore et al., 2006; Stouffer and Yoder, 2011; Shoji et al., 2016), supporting the view that connectivity decreases in specific functional brain systems may be associated with particular behavioral changes (Andrews-Hanna et al., 2007; Onoda et al., 2012).

Even more remarkable is the fact that within high-order cognitive networks young to middle-age FC decline is more pronounced than the changes observed between middle-aged and older adults. While this finding conforms with earlier structural connectivity findings (Salat et al., 2005; Giorgio et al., 2010) as well as with age-related alterations in genetic, biochemical, and neurophysiological variables (Antonini et al., 1993; Kuhn et al., 1996; Mozley et al., 1996; Sheline et al., 2002; Lu et al., 2004; Hattiangady et al., 2005; Rex et al., 2005; Lynch et al., 2006; Hamilton et al., 2013; Aliper et al., 2015), it appears inconsistent with the progressive course of cognitive decline. Although the source for this lifespan FC dynamics cannot be determined by the current study, compensation and dedifferentiation theories might be proposed to account for the apparent late FC plateau. Park and Reuter-Lorenz (2009) suggested in their “scaffolding theory of aging and cognition” (STAC) that although the brain is subjected to multiple neural challenges throughout the lifespan, adaptive compensatory processes help it maintain homeostatic cognitive function up to a certain point. The absence of further FC decline and even connectivity increment in several node pairs in older age might be related to such compensatory mechanisms, as prudent observation of a few behavioral studies seems to reveal a hitherto unreported cognitive plateau around middle-age (Villardita et al., 1985; Park et al., 1996; Buckner, 2004; Park and Payer, 2006). Dedifferentiation, the loss of functional specialization in brain activity, is an alternative theory commonly proposed to account for cognitive aging (Park and Reuter-Lorenz, 2009). One model which was proposed to represent dedifferentiation is the hemispheric asymmetry reduction in older adults (HAROLD) (Cabeza et al., 2002; Dolcos et al., 2002; Li et al., 2009). In line with this model and in accordance with our findings, Zuo et al. (2010) showed that FC between geometrically corresponding interhemispheric regions follows a quadratic U-shaped trajectory across the lifespan with a turning point in middle-age in heteromodal regions. Future insights into the physiological origins of BOLD-based FC and their age-related changes (e.g., dopaminergic transmission; Ferreira and Busatto, 2013) as well as future studies concurrently evaluating FC and cognitive performance may inform on the underlying mechanisms of the lifetime FC variations observed here.

In addition to temporal aspects of connectivity decline, spatial features should be considered. Our study supports the view that different brain systems are differentially affected by aging. Here, the MN showed a significantly different FC time course compared with high-order cognitive networks (early FC increment followed by a late decrement). Age-related changes in FC within the MN have been studied only rarely and results are conflicting with both decreases (Wu et al., 2007) and increases (Tomasi and Volkow, 2012; Solesio-Jofre et al., 2014) reported. However, consistent with our finding, a previous analysis of brain structural covariance networks (Li et al., 2013) uncovered a difference between motor and high-order cognitive networks. Li et al. (2013) report that non-motor networks demonstrate a distributed topology in the young group, shrinkage into a more localized topology in the middle-aged group, and maintained localized topology in the older group, while the MN shows increased spatial distribution in middle-age and decreased distribution in older participants. Moreover, a recent study by Song et al. (2014) reported an equivalent opposing effect of aging on FC within the DMN and a sensorimotor network.

The observed difference between the motor and high-order cognitive networks is in line with the “last in, first out” hypothesis. Moreover, differential vulnerability to aging is also noted within the same network. Interestingly, the vmPFC, LTC and frontal pole, here showing maximal connectivity reduction among DMN regions, were previously reported as being regions of advanced age-related atrophy and greater expansion during evolution (Hill et al., 2010; Bludau et al., 2014; Fjell et al., 2014). As for the MN, the reduced connectivity in an anterior cerebellar region, probably associated with motor function (Bernard et al., 2013), is consistent with previous reports of reduced cortico-cerebellar connectivity with aging (Bernard et al., 2013; Bernard and Seidler, 2014). The concurrent increased connectivity in cortical MN regions is consistent with the hypothesis that shorter connections are enhanced in older individuals, while longer connections are diminished (Rowe et al., 2006). Other early changes observed in the MN are probably due to increased anticorrelations of subcortical regions with the LMC.

Age-related alterations in interrelations between networks represent another central finding of the current study. Most prominent alterations were increased correlations (e.g., DAN-MN) and decreased anticorrelations (e.g., DMN-SN) between networks in the transition from young to middle adulthood. Our observations strongly support accumulating evidence for enhanced communication between functional networks with aging (Chan et al., 2014; Geerligs et al., 2015; Turner and Spreng, 2015; Gallen et al., 2016; Spreng et al., 2016; Tsvetanov et al., 2016). Increased correlations and decreased anticorrelations between networks both reflect reduced selectivity and specificity in the brain’s intrinsic functional architecture. These changes have been associated with compromised cognitive performance (Kelly et al., 2008; Hampson et al., 2010; Antonenko and Floel, 2014; Chan et al., 2014; Tsvetanov et al., 2016) and hence comply with the dedifferentiation theory of aging; yet, a role in compensation has also been suggested (Gallen et al., 2016; Grady et al., 2016). It should be mentioned that GSR used as a preprocessing step in our study has been reported to artifactually enhance anticorrelations between networks (Fox et al., 2009; Murphy et al., 2009). However, a repeated analysis without GSR confirmed age-related reduced anticorrelation between the DMN and SN (Supplementary Figure S3). This finding is consistent with recent aging studies omitting GSR (Keller et al., 2015; Spreng et al., 2016) and supports previous literature suggesting neural rather than artifactual origin of anticorrelations (Chang and Glover, 2009; Fox et al., 2009; Carbonell et al., 2011; Chai et al., 2012; Keller et al., 2013; Power et al., 2014). Additionally, in line with a few previous reports (Allen et al., 2011; Onoda et al., 2012), our analysis also reveals diminished between-network connectivity with aging for some network pairs. Undoubtedly, the complexity of age-related changes in integration of information between networks warrants further investigation. The importance of the present findings is in pointing out decreased segregation of brain systems by middle-age.

The use of a dataset such as the ‘1000 Functional Connectomes’ Project inherently imposes a source of variability among participants in terms of demographic parameters, cognitive intactness, arousal level, eye opening condition, head movement and technical acquisition parameters, features that have been reported to affect resting-state correlations (Power et al., 2012; Van Dijk et al., 2010, 2012). The statistical analysis was designed to account for these potential biases, as far as the data allowed. Inclusion of participants of different age groups further augments variability in terms of resting metabolic rate (Peng et al., 2014), resting cerebral blood flow (Lu et al., 2011), vascular CO_2_ reactivity (Lu et al., 2011; Murphy et al., 2013); blood pressure (AlGhatrif et al., 2017); and hemoglobin concentration (Patel, 2008) all known to affect BOLD fMRI signal and/or the coupling of neural activity to the BOLD signal (D’Esposito et al., 1999; Levin et al., 2001; Patel et al., 2012; Murphy et al., 2013; Mark et al., 2015). Nevertheless, the uneven effect of aging on large-scale brain networks (e.g., DMN vs. MN), emphasized here and elsewhere (Song et al., 2014; Marstaller et al., 2015), probably indicates that age-related FC alterations cannot be merely attributed to changes in neurovascular coupling. Moreover, the GSR step applied in data preprocessing is expected to lessen the confounding effects of the above mentioned physiological factors as well as of cardiac and respiratory cycles (Van Dijk et al., 2010), which have not been directly measured. Though a matter of long-standing debate (Fox et al., 2009; Murphy et al., 2009; Murphy and Fox, 2016), GSR seemed warranted under the current study due to several important reasons: it was shown to enhance the detection of network-specific seed-based correlations (Fox et al., 2009), which are at the core interest of this work; reduce motion and hardware artifacts (Power et al., 2016), an important advantage when evaluating a large multi-center dataset; and enhance the neuronal-hemodynamic correspondence (Keller et al., 2013). Taking into consideration the interpretive complexity imposed by GSR (Murphy and Fox, 2016), we repeated the analyses without GSR, confirming that the study’s main outcomes are present. Another limitation of this study is the absence of data regarding WM hyperintensity burden which has been shown to increase with age (Nyquist et al., 2015) and affect FC (Liang et al., 2016). As both FC decrements and increments have been reported in the presence of WM lesions (Liang et al., 2016), it is difficult to appreciate this parameter’s influence on our results. Finally, the absence of cognitive data for this cohort limits the ability to contemplate regarding the mechanisms underlying current observations. Future single-site longitudinal studies combining detailed analysis of behavioral performance, elaborated demographic information, laboratory data, structural and functional imaging data, as well as measures to control for non-neuronal physiological parameters, might better address the above mentioned confounds and expand the scope of our findings.

In summary, our results confirm the hypothesis that normal brain aging involves reorganization of large-scale functional brain systems. Furthermore, it underscores the early occurrence of FC alterations where high-order cognitive networks are involved. In an era in which we seek ways of augmenting cognition throughout healthy aging, deferring the onset of connectivity decline, slowing down its pace and prolonging the proposed subsequent plateau should be considered potential targets for intervention.

## Ethics Statement

The imaging data of the current study were obtained from 17 research sites through the online dataset of the International Neuroimaging Data-sharing Initiative (INDI). Each center’s ethics committee approved submission of de-identified data as detailed in the NITRC: ‘1000 Functional Connectomes’ Project site (http://fcon_1000.projects.nitrc.org/). The institutional review board of Rambam healthcare campus approved the receipt and analysis of these data.

## Author Contributions

TS-T, NB, AE, JA-P, and IK contributed to the conception and design of this work. TS-T, NB, and IK collected, analyzed, and interpreted the data. RP contributed to data analysis. ES designed and performed the statistical analyses. TS-T, NB, ES, JA-P, and IK contributed to in-depth discussions about methods and results. TS-T and IK wrote the paper. All authors revised and approved the manuscript.

## Conflict of Interest Statement

The authors declare that the research was conducted in the absence of any commercial or financial relationships that could be construed as a potential conflict of interest. The reviewer NE and handling Editor declared their shared affiliation, and the handling Editor states that the process nevertheless met the standards of a fair and objective review.

## Footnotes

1 http://fcon_1000.projects.nitrc.org/

2 http://surfer.nmr.mgh.harvard.edu/
