# Supplementary material for: Early Age-Related Functional Connectivity Decline in High-Order Cognitive Networks
Source: Front Aging Neurosci. 2017 Jan 10;8:330. doi: 10.3389/fnagi.2016.00330 (PMC5223363; doi:10.3389/fnagi.2016.00330)
Supplement: Supplementary file 1 [file Data_Sheet_1.PDF]

Supplementary Material

Early Age-Related Functional Connectivity Decline in High-Order Cognitive Networks

Siman-Tov Tali[1*], Bosak Noam[2], Sprecher Elliot[3,4], Paz Rotem[1], Eran Ayelet[5], Aharon-Peretz Judith[1,4], Kahn Itamar[2*]

*** Correspondence:**
Tali Siman-Tov
simantov.tali@gmail.com

Itamar Kahn
kahn@technion.ac.il

Supplementary Figures

Figure S1. Participants' distribution by age group and center.

Figure S2. Difference matrices between age groups with and without GSR.

Difference matrices of young vs. middle-aged (Y-M) and middle-aged vs. old (M-O) participants (entire cohort, n=887) are shown for each network, for both analyses, with and without GSR. Rows and columns of matrices denote the ROIs that were chosen to represent each network (see Table 3). Matrix entries represent the age group difference in connectivity strength (*z(r)* value) for each node pair. Entries marked by a dot survived FDR correction for multiple comparisons. High-order cognitive networks show more pronounced connectivity decline in the transition from Y to M than from M to O. This finding remains after removing GSR for the DMN, SN and FPCN. The MN mostly shows early connectivity increments and late connectivity decrements, either with or without GSR. The AN and VN show reductions in both transitions: from Y to M and from M to O; however, without GSR less pronounced reduction is observed in the Y to M transition of the AN.

Figure S3. Age-related internetwork connectivity changes computed without GSR

Internetwork connectivity matrices of the young (Y), middle-aged (M) and old (O) groups (*upper row*). Rows and columns denote the ROIs that were chosen to represent each network (see Table 3). Matrix entries represent connectivity strength between each two nodes (*z(r)* value). Difference matrices of the Y vs. M and M vs. O groups (*lower row*). Matrix entries represent age-group difference in connectivity strength for each node pair. Entries marked by a dot survived FDR correction for multiple comparisons. Similar to the analysis with GSR, most significant age-related changes in internetwork connectivity occurred in the transition between Y and M groups. Though GSR removal induced a general increase in internetwork correlations, the direction of change between age groups was mostly kept.
