# Supplementary figures and images for: Early Age-Related Functional Connectivity Decline in High-Order Cognitive Networks
Source: Front Aging Neurosci. 2017 Jan 10;8:330. doi: 10.3389/fnagi.2016.00330 (PMC5223363; doi:10.3389/fnagi.2016.00330)

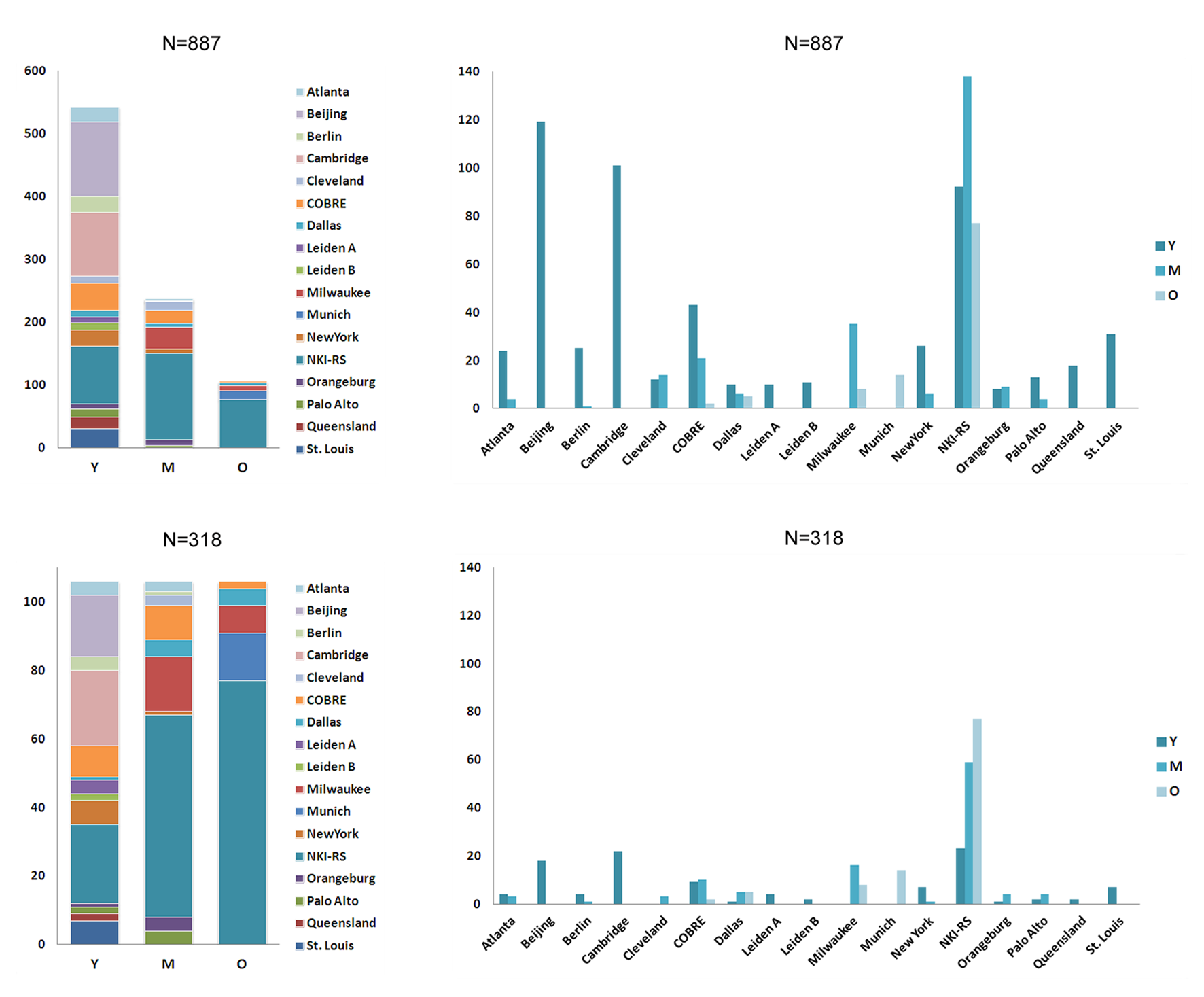

Supplement: Supplementary file 2 [file Image_1.TIF]

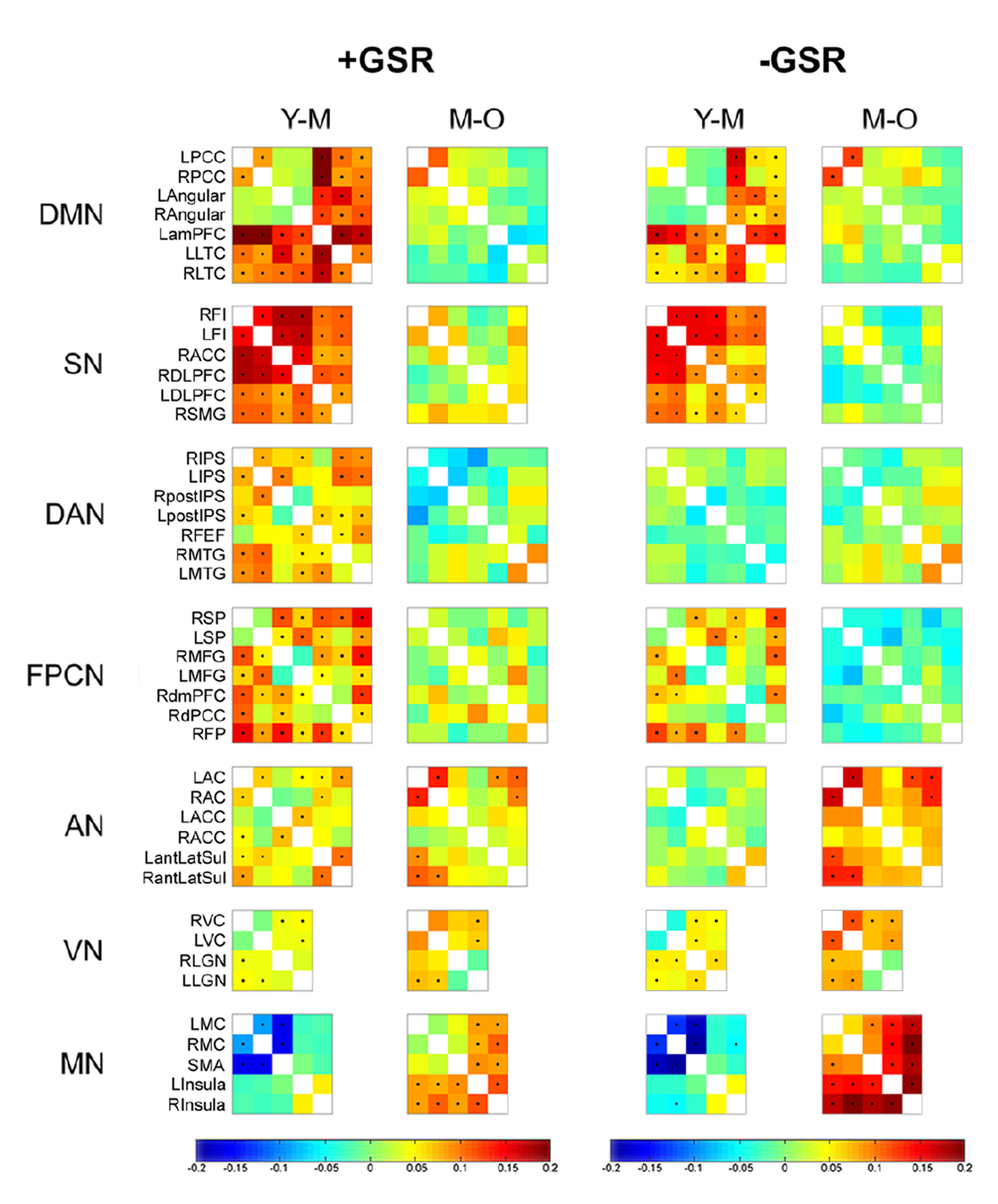

Supplement: Supplementary file 3 [file Image_2.TIF]

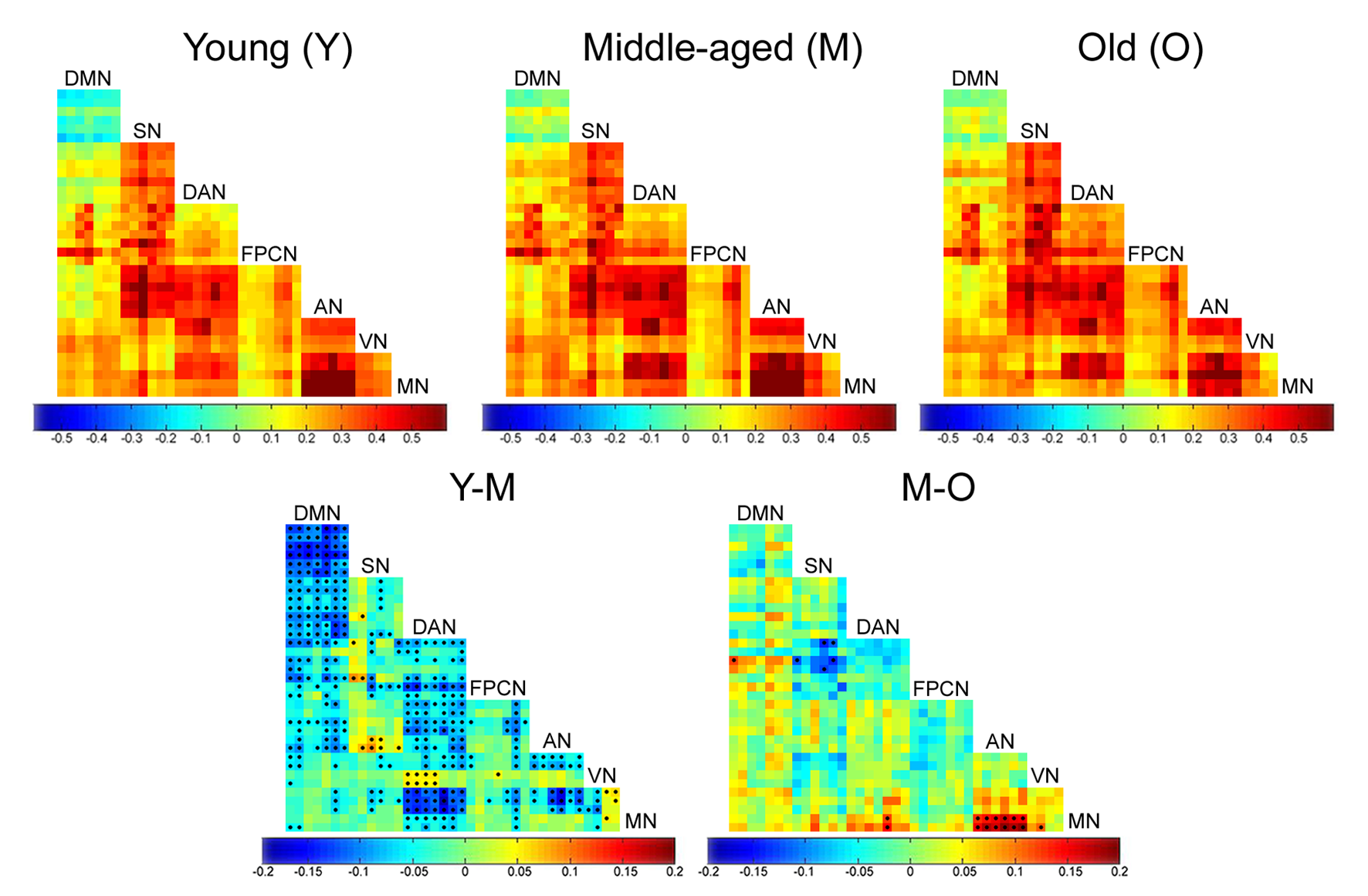

Supplement: Supplementary file 4 [file Image_3.TIF]
